# Assessment of Validity and Reproducibility of the Zinc-Specific Dietary Intake Questionnaire Conducted for Young Polish Female Respondents

**DOI:** 10.3390/nu10010104

**Published:** 2018-01-19

**Authors:** Dominika Głąbska, Aleksandra Staniec, Dominika Guzek

**Affiliations:** 1 Department of Dietetics, Faculty of Human Nutrition and Consumer Sciences, Warsaw University of Life Sciences (WULS-SGGW), 159c Nowoursynowska Str., 02-776 Warsaw, Poland; aleksandra_staniec@sggw.pl; 2 Department of Organization and Consumption Economics, Faculty of Human Nutrition and Consumer Sciences, Warsaw University of Life Sciences (WULS-SGGW), 159c Nowoursynowska Str., 02-776 Warsaw, Poland; dominika_guzek@sggw.pl

**Keywords:** zinc, validation study, ZINC-FFQ, food frequency questionnaire, validity, reproducibility

## Abstract

One of the brief methods enabling the assessment of the zinc intake and identification of individuals characterized by insufficient zinc intake, is zinc-specific food frequency questionnaire. The aim of the study was to assess the validity and reproducibility of the elaborated zinc-specific food frequency questionnaire ZINC-FFQ (Zinc INtake Calculation—Food Frequency Questionnaire) in a group of young Polish female respondents. The validity was assessed in comparison with 3-day dietary records, while reproducibility was assessed for the ZINC-FFQ filled in twice (FFQ1 and FFQ2—six weeks apart). Bland–Altman indexes in the assessment of validity were 5.5% (FFQ1) and 6.7% (FFQ2), while in assessment of reproducibility it was 3.3%. In the assessment of reproducibility, 83% of respondents were classified into the same category of zinc intake adequacy and 72% of respondents were classified into the same tertile, that contributed to weighted *κ* statistic of 0.65 (substantial agreement). It may be concluded, that ZINC-FFQ is characterized by a validity on a satisfactory and reproducibility on a very good level, in a group of young Polish female respondents, and may be applied to indicate individuals characterized by the risk of insufficient intake.

## 1. Introduction

In spite of the fact that severe zinc deficiency is rare, according to the World Health Organization (WHO) [1], mild-to-moderate zinc deficiency is commonly stated throughout the world. Each third individual of the world population is affected by its deficiency of various severities, while in some regions the frequency is even 73% [2]. Each year, in the global population of children under five years of age, zinc deficiency results in about 800 thousand of deaths, that are attributed to diarrhea, pneumonia and malaria [3], as zinc intake reduces related mortality [4].

The zinc delivered during pregnancy results in fetal accumulation and newborn zinc status, as well as zinc intake during lactation results in child zinc status, that was proven in the systematic review and meta-analysis of Petry et al. [5] for low-dose of supplementation. Taking it into account, it must be indicated, that for zinc status in children, the maternal zinc intake, both from diet and supplementation, may be crucial. In the systematic review of Chaffee and King [6], it was indicated, that zinc supplementation in pregnant women may contribute to the reduced risk of the preterm birth, but the currently available information does not support the routine use of zinc supplementation during pregnancy [7], unless emergency supplementation in the low-income countries is needed [8].

Despite the fact, that on the basis of the food supply, it is estimated, that zinc deficiency risk is decreasing [9], the prevalence rate of inadequate zinc intake for general population of Central and Eastern Europe is still 10% [10]. It indicates the need for appropriate interventions, that should be implemented both in the developing and developed countries, in groups of young women characterized by the risk of zinc deficiency [11]. 

The pregnant and lactating women zinc requirement is associated with the amount of zinc retained in newly accrued tissues and the zinc secreted in breast milk [12], but the education should include both pregnant or lactating women, and those in preconception period, in order to prevent the deficiency during pregnancy. Among the methods of intervention allowing to educate and change the nutrient intake, are food frequency questionnaires [13]. However, due to the lack of the available validated brief questionnaires enabling rapid assessment of the zinc intake in European countries, there is a need to elaborate and validate such questionnaire.

The aim of the study was to assess the validity and reproducibility of the elaborated zinc-specific food frequency questionnaire ZINC-FFQ (Zinc INtake Calculation—Food Frequency Questionnaire) in a group of young Polish female respondents.

## 2. Materials and Methods

The study was approved by the Bioethical Commission of the National Food and Nutrition Institute in Warsaw (No. 0701/2015), while it was conducted in compliance with the guideline statements of the Declaration of Helsinki.

### 2.1. Designing the ZINC-FFQ

The designed ZINC-FFQ included products being sources of zinc (content of 0.01 mg per 100 g or higher [14]), while other products were not included, due to the fact, that it was to be a brief questionnaire to estimate the zinc intake, but not the intake of the other nutrients. All food products included in the designed questionnaire were clustered into 13 groups and 46 food product items, while the typical serving sizes were presented in grams and described [15], as the same approach was applied as in the previous studies [16,17,18]. To not influence the answers of respondents, the elaborated ZINC-FFQ (Table 1) did not contain the zinc content information that was presented only in the questionnaire calculation key (Table 2). 

In the ZINC-FFQ, respondents were asked about the number of servings of food items consumed typically during a week, while both products consumed separately and as an element of the recipe of dishes were to be included and decimal parts were allowed. The same approach to calculate the typical daily zinc intake was applied as in the previous questionnaires [16,17,18].

### 2.2. The ZINC-FFQ Validation Procedure

**Figure 1:**
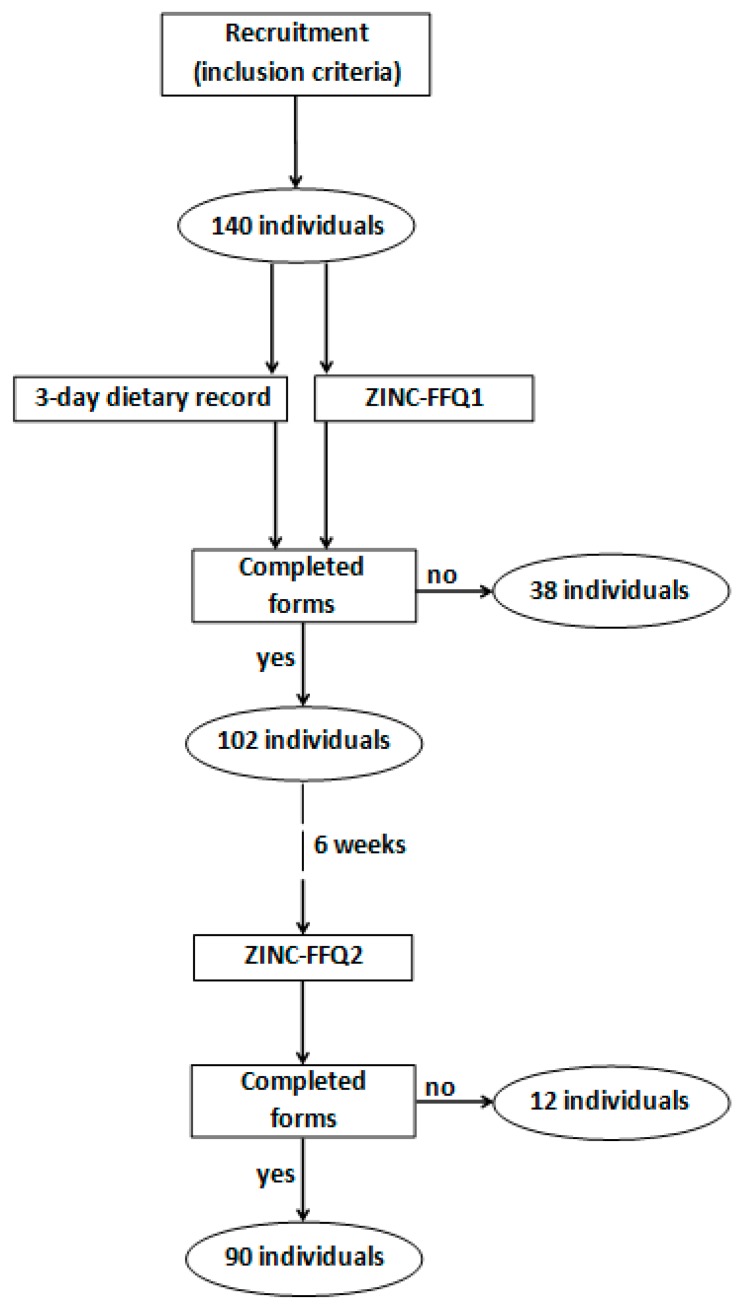
Participant recruitment to the validation of the questionnaire. ZINC-FFQ = Zinc INtake Calculation—Food Frequency Questionnaire.

For the validation of the ZINC-FFQ, the same approach was applied as in previous studies [16,17,18]. The convenience sampling of women living in Warsaw was applied. The validation was conducted in the same group of young Polish female respondents, as the validation of IOdine Dietary INtake Evaluation-Food Frequency Questionnaire (IODINE-FFQ) designed to assess the iodine intake [18], so the inclusion criteria, exclusion criteria, characteristics of respondents and recruitment procedure (Figure 1) as well as validation approach [19] were previously presented [18].

According to the Biomarkers of Nutrition for Development (BOND) Zinc Expert Panel [20], the assessment of the dietary zinc intake is one of the recommended methods to assess the zinc status. Moreover, the ZINC-FFQ was planned to be validated against the other method enabling calculation of intake on the basis of the self-reported data, so the 3-day dietary record was chosen as the reference method. The record was applied according to the same rules as in the previous studies [16,17,18], while the zinc intake was analyzed using the Polish database of the nutritional value of products [14], applying Polish dietician software.

### 2.3. Statistical Analysis

The statistical approach included assessment of validity (while results of the ZINC-FFQ1 and ZINC-FFQ2 were compared with the results of the 3-day dietary record) and of reproducibility (while results of the ZINC-FFQ1 and ZINC-FFQ2 were compared). The analysis included Bland–Altman plot, tertiles distribution, weighted *κ* statistic, adequacy assessment in comparison with Estimated Average Requirement (EAR) level of 6.8 mg [21], as well as supplementary methods, while analysis was conducted as in the previous study [22].

## 3. Results

Zinc intake observed in the group of young female respondents is presented in Table 3. While using both methods it was stated that the majority of young female individuals were characterized by adequate zinc intake (higher than the EAR level of 6.8 mg per day).

The validation of the ZINC-FFQ including tertiles distribution, weighted *κ* statistic and adequacy assessment are presented in Table 4. In comparison with 3-day dietary record, over 65% of young female respondents were classified in the same category of zinc intake adequacy and less than 50% were classified into the same tertile. At the same time, in the comparison between ZINC-FFQ1 and ZINC-FFQ2, over 80% of young female respondents were classified in the same category of zinc intake adequacy and almost 75% were classified into the same tertile, that contributed to weighted *κ* statistic of 0.65 (substantial agreement).

**Figure 2:**
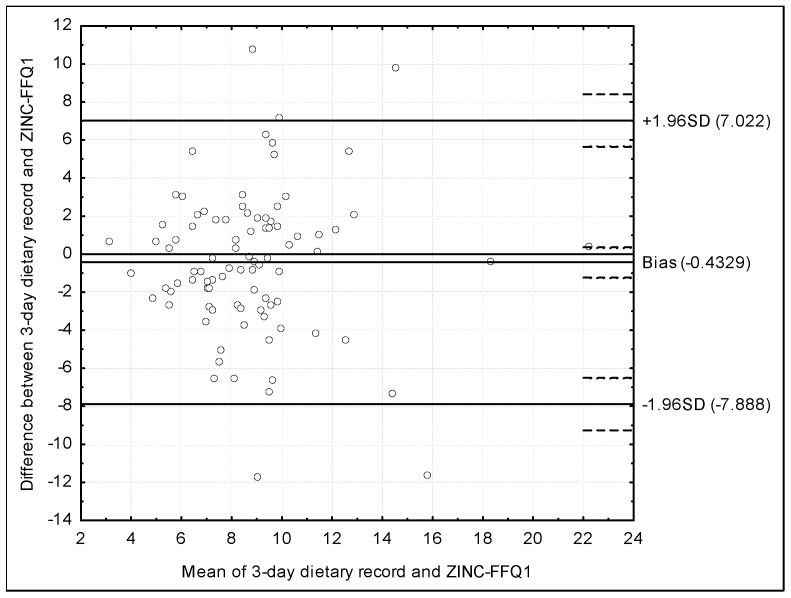
The assessment of the validity of the ZINC-FFQ conducted using the Bland–Altman plot for the ZINC-FFQ1 (Bland–Altman index of 5.5%). ZINC-FFQ1 = first food frequency questionnaire. SD = Standard deviation.

The assessment of the validity of the ZINC-FFQ conducted using the Bland–Altman plot for the ZINC-FFQ1 is presented in Figure 2. The mean absolute difference of the zinc intake was 0.4329, while the agreement limit was observed to be from −7.022 to 7.888 and a Bland–Altman index of 5.5% was obtained.

**Figure 3:**
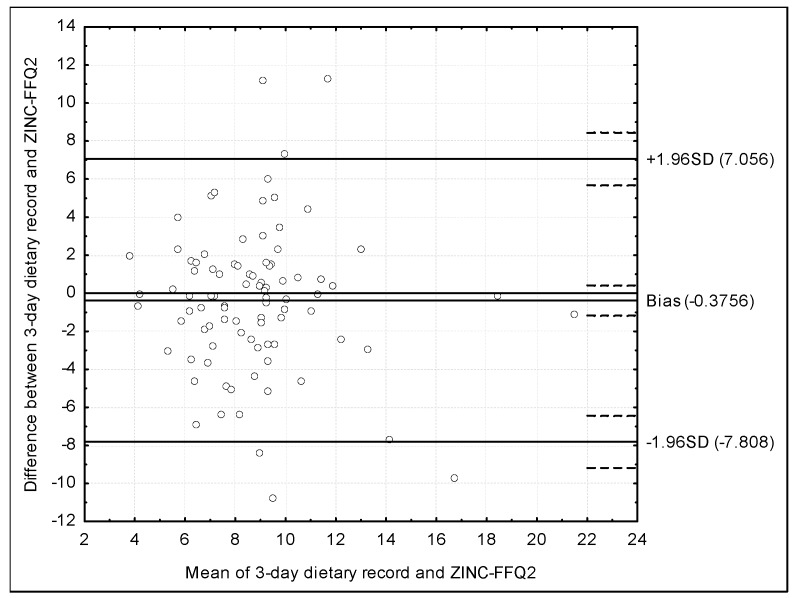
The assessment of the validity of the ZINC-FFQ conducted using the Bland–Altman plot for the ZINC-FFQ2 (Bland–Altman index of 6.7%). ZINC-FFQ2 = second food frequency questionnaire.

The assessment of the validity of the ZINC-FFQ conducted using the Bland–Altman plot for the ZINC-FFQ2 is presented in Figure 3. The mean absolute difference of the zinc intake was 0.3756, while the agreement limit was observed to be from −7.056 to 7.808 and a Bland–Altman index of 6.7% was obtained.

**Figure 4:**
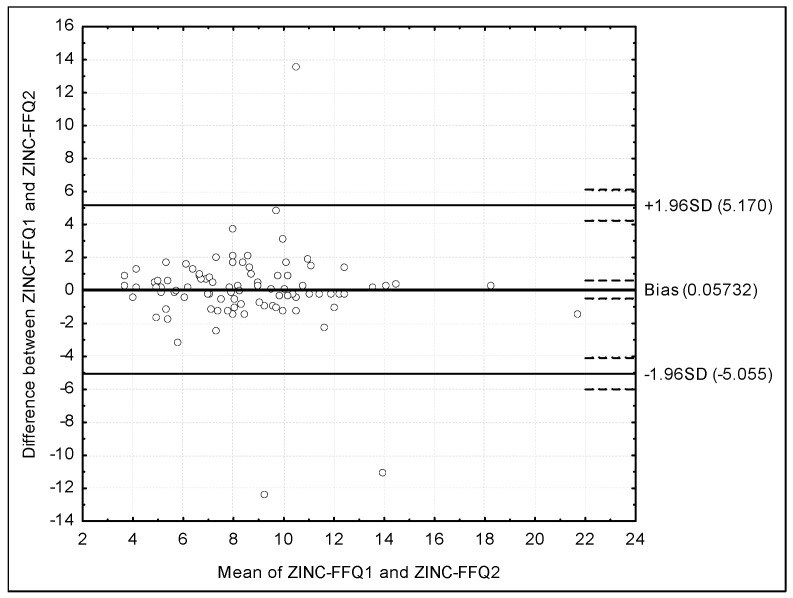
The assessment of the reproducibility of the ZINC-FFQ conducted using the Bland–Altman plot (Bland–Altman index of 3.3%). ZINC-FFQ1 = first food frequency questionnaire. ZINC-FFQ2 = second food frequency questionnaire.

The assessment of the reproducibility of the ZINC-FFQ conducted using the Bland–Altman plot is presented in Figure 4. The mean absolute difference of the zinc intake was 0.05732, while the agreement limit was observed to be from −5.055 to 5.170 and a Bland–Altman index of 3.3% was obtained.

In the assessment of reproducibility, the Root Mean Square Errors of Prediction (RMSEP) of zinc intake estimation in a group of young women was 2.44 mg, the Median Absolute Percentage Errors (MdAPE) was 9.98%, while the correlation was significant (*p* = 0.0000; *R* = 0.7440).

## 4. Discussion

The most important consequences of zinc deficiency include the adverse fetal effects, as well as increased newborn [7] and children death rate [3]. However, the influence on the offspring is not the only effect proven for young women, as in the systematic review of Lomagno et al. [23], conducted for young and premenopausal women, the better zinc status was indicated as associated with improved emotional and cognitive functioning. Taking it into account, not only pregnant women and those in preconception period would benefit from the control of the zinc intake, but also those who do not intend to be pregnant. 

### 4.1. Food Frequency Questionnaires for Pregnant Women

A number of applied multiple-nutrients food frequency questionnaires include zinc intake assessment, while some of them were designed for a specific group of pregnant women. Such pregnancy questionnaires were validated e.g., in United States of America [24,25], China [26,27], Brasil [28], or Japan [29], but also in European countries—Great Britain [30], Norway [31], and Spain [32].

It is indicated by authors that the intake during pregnancy changes significantly—it may change within each trimester, as well as it is in general less stable than in the case of non-pregnant women, so a significant day-to-day variation is observed [24]. It may result from the appetite fluctuation, caused by nausea or vomiting, changing food preferences, as well as energy requirements [27]. As the diet during pregnancy may change, the season of the year when women is pregnant is also important, as the summer to autumn period is indicated as characterized by the highest seasonal variation, resulting from the fruit and vegetables intake [26]. 

Regarding the above-mentioned, it must be emphasized that a constant monitoring of intake during pregnancy is needed and food frequency questionnaires enabling assessment of multiple nutrients may be a good option. The dietary record may not be a better method than food frequency questionnaire, as Brantsæter et al. [31] indicated that in their study pregnant women were omitting snacks which were considered as unhealthy, in spite of the fact, that they were asked not to change their typical dietary habits.

However, the indicated questionnaires were designed to assess the intake of many nutrients and, as a result, many questions are included. Consequently, it is indicated, that in the case of validation conducted by Brunst et al. [25], it took about 20–30 min to complete the interviewer-administrated modified Block98 Food Frequency Questionnaire [33]. As a result, while only the zinc intake is to be assessed, rather a zinc-specific brief questionnaire should be applied.

### 4.2. Food Frequency Questionnaires Assessing Zinc Intake

In the review of Serra-Majem et al. [34] it was indicated, that for the zinc assessment, applied using food frequency questionnaires, the methods analyzing intake, such as records and recalls, had an acceptable correlation with validated food frequency questionnaires. Simultaneously, correlations were increased while weighted dietary records were applied and while the assessment of applied supplementation was included [34].

At the same time, the validations conducted using biomarkers often are not so positive. For the electronic semi-quantitative food frequency questionnaire, including 235 items, adapted from the Blue Mountains Eye Study Food Frequency Questionnaire [35], assessing inter alia zinc intake, it was observed, that between zinc intake and serum zinc concentration, there was no significant trend [36]. The lack of association between zinc intake assessed using food frequency questionnaire and zinc biomarkers was explained by many authors. Fayet et al. [36], interpreted it as resulting from the number of other dietary factors that affect zinc absorption [37]. Simultaneously, Brunst et al. [25] indicated the fact that serum zinc concentration reflects the recent intake from food products and supplementation, while the intake from questionnaire reflects the typical intake from diet and sometimes also from supplementation. Moreover, Brantsæter et al. [31], validating food frequency questionnaire for pregnant women in the Norwegian Mother and Child Cohort Study (MoBA) indicated that just a few biological markers are directly related to the nutrients intake, so biomarkers use is limited, mainly due to the high cost [38]. In the conducted study, the blood samples were not collected, as the aim of the study included validation in comparison with the other method of the dietary intake assessment, but it must be indicated, that in the following analysis, the validation in comparison with biomarkers of the zinc status will be needed.

In the review analyzing assessment methods for zinc intake [34], the correlation coefficients for the food frequency questionnaires, validated using methods assessing the dietary intake, were analyzed and it was indicated, that the correlation coefficients for zinc depend on the food items number in food frequency questionnaires. It was stated, that the number of food items lower than 100 is associated with a slightly higher values of correlation coefficient than for higher number of items [34].

The length of the form and a number of questions may be a crucial issue and, as was revealed by Serra-Majem et al. [34], the lower number of questions in the form may be not only easier for respondents, but also associated with a higher validity. Regarding the above-mentioned, the constant analysis of questionnaires is necessary, while redundant food items should be excluded to simplify the questionnaire [39].

For the zinc, it was observed by Samman et al. [40], in the study conducted in Australia, that assessed the validity of the short food frequency questionnaire while the various number of food products from the questionnaire were included into calculation. In the indicated study, it was observed, that the number of food product items in the questionnaire may be successfully reduced from 74 to 37 (food products contributing to 80% of the total zinc intake), as the similar results in the validation were still observed [40].

### 4.3. Validations Conducted Using Bland–Altman Index

The Bland–Altman plot is recommended to be used instead of the other methods of assessment, in spite of the fact, that in practice it is applied by researchers less often than the other methods [13]. In the present study, the assessment of validity and reproducibility of the ZINC-FFQ, conducted using the Bland–Altman plot indicates a positive validation, or borderline positive validation, on the basis of the commonly applied criteria [41]. In general, a Bland–Altman index of 5% is indicated as a borderline [41]. Such level of a maximum of 5% was observed for the ZINC-FFQ in the assessment of reproducibility (3.3%), while for the validity, the level of 5.5% was observed for FFQ1, but also 6.7%—for FFQ2. The lower validity level for FFQ2 may be associated with the fact, that FFQ2 was filled in 6 weeks after the FFQ1, while FFQ1 was filled in at the similar time as the 3-day dietary record. 

However, other authors validating their brief food frequency questionnaires to assess the zinc intake, often observe even higher levels of Bland–Altman index, but they still interpret the questionnaires as a good methods in the assessment. Samman et al. [40], analyzing a food frequency questionnaire of a various number of items, administrated during a personal interview, in a group of 22 young women, being validated against a 7-day weighted dietary record, for the number of 37-item, indicated the successful validation. The number of 20 individuals, out of 22, within the limit of agreement, indicated a Bland–Altman index of 9.1%, being both higher than 5% indicated as a borderline in the assessment [41] and higher than Bland–Altman indexes observed in the validation of ZINC-FFQ in the present study. 

Similarly, the Bland–Altman plot was analyzed in the study of Alsufiani et al. [42], in the assessment of the 64-item food frequency questionnaire adapted from the questionnaire previously validated by Samman et al. [40], by adjusting food products list to the nutritional habits in the population of Saudi Arabia. The number of 95 individuals, out of the diverse population of 100, within the limit of agreement, both in the assessment of reproducibility and validity, indicated a Bland–Altman index of 5.0%. The indicated Bland–Altman index may be interpreted as a borderline significant, that was confirmed by authors, who stated, that the reasonable validity and a high repeatability was observed [42]. 

In spite of the fact that the validity observed in the present study (especially in the case of FFQ2) was lower than recommended, the reproducibility of 3.3% was higher than for the indicated studies of Samman et al. [40] and Alsufiani et al. [42]. As a result, it may be supposed, that ZINC-FFQ may be particularly useful in the repeated measurements, while the researcher may benefit from the high reproducibility.

The observed higher reproducibility than in the mentioned study [42] may result from the different procedure chosen during designing the questionnaire. In the study of Samman et al. [40] food items of a zinc content no lower than 0.5 mg/100 g were chosen and afterwards, during analysis, they removed food product items, until they obtained 37 items contributing 80% of total zinc intake in the assessed group. Alsufiani et al. [42] adapted the questionnaire previously validated by Samman et al. [40], so the procedure was similar. At the same time, in the present study of the ZINC-FFQ, food items characterized by zinc content of 0.01 mg per 100 g or higher were chosen and afterwards they were clustered into 46 food product items. Such procedure may result in a higher accuracy of estimation, as no products were removed from the questionnaire during the designing procedure.

### 4.4. Validations Conducted Using Other Methods

In spite of the fact, that the Bland–Altman plot is the major method in the validation of questionnaires, it is allowed to use the *κ* statistic in the assessment of reproducibility and validity, for a small numbers of ordered categories [13]. It is very rarely applied by researchers, but in the present study of the ZINC-FFQ, it confirmed the substantial agreement in the case of reproducibility and a slight agreement (values lower than 0.20) in the assessment of validity. 

Authors more often use the analyses of the tertiles or quartiles distribution and adequacy assessment. According to the criteria of Masson et al. [43], when more than 50% of individuals are equally classified, using the compared methods, and less than 10% of individuals are classified into opposite categories, the validity is confirmed. In the present study, the criteria were fulfilled in the assessment of reproducibility of ZINC-FFQ (72.22% correctly classified and 3.33% grossly misclassified), but not in the assessment of validity (43–47% and 16–20% correctly classified and grossly misclassified, respectively, for FFQ1 and FFQ2). It confirms as previously indicated a very good reproducibility, but a minor validity.

The criteria of Masson et al. [43] were fulfilled in the previously mentioned assessment conducted by Alsufiani et al. [42], as in the comparison with the 3-day dietary record for zinc intake, 62% of individuals were correctly classified into tertiles and 2% were grossly misclassified, that confirmed a positive validation. The criteria were also fulfilled in the assessment conducted by Heath et al. [44], while the Meal-Based Intake Assessment Tool (MBIAT) was analyzed (consisting of 630 food products sorted into 16 groups) in healthy male individuals and 60% of individuals were correctly classified into quartiles, while there was no grossly misclassified individuals.

However, similarly as in the case of the Bland–Altman index, in other validation studies are also observed values, that do not fulfill the criteria of Masson et al. [42]. In the study of Fayet et al. [36], in the assessment of electronic semi-quantitative food frequency questionnaire, including 235 items, in young female adults, in comparison with the results of zinc intake obtained on the basis of repeated 24-h dietary recall, 26.4% of individuals were correctly classified into quartiles and 11.3% were grossly misclassified, while questionnaire was interpreted as a relatively valid. 

The criteria of Masson et al. [42] are also formulated for the analysis of correlation and it is indicated, that while the correlation coefficient is higher than 0.5, the validity is confirmed. In the present study of ZINC-FFQ, it was analyzed and confirmed in the assessment of reproducibility (*p* = 0.0000; *R* = 0.7440). Similarly, it was confirmed in the reproducibility assessment of the questionnaire conducted by Heath et al. [44] (*R* = 0.64 and *R* = 0.73, for various periods between repeated measurements), as well as in the assessment of validity of the questionnaire conducted by Samman et al. [40] (*p* < 0.001; *R* = 0.81 and *p* < 0.001; *R* = 0.76 for 74-item and 37-item questionnaire, respectively).

However, similarly as in the previously indicated cases of the Bland–Altman index and analysis of tertiles/quartiles, in other validation studies are also observed values that do not fulfill the criteria of Masson et al. [43]. It was observed in the study of Alsufiani et al. [42], that the criteria of Masson et al. [43] were fulfilled in the assessment of reproducibility (*R* = 0.758), but not in the assessment of validity (*R* = 0.410), for the questionnaire interpreted as characterized by a high repeatability and a reasonable validity. Similarly, in the study of Lacey [45], for a Zinc Assessment Tool (ZAT), validated in United States of America, in male and female students, the validity was observed, while compared with the 3-day dietary record, only for female (*p* < 0.001; *R* = 0.30), but not for male respondents (*R* = 0.21), while for both cases correlation coefficient was lower than 0.5.

## 5. Conclusions

In comparison with the validation studies of other zinc-specific food frequency questionnaires, for the ZINC-FFQ an average validity, but a better reproducibility were observed. It may be concluded, that ZINC-FFQ is characterized by a satisfactory level of validity and very good reproducibility in young Polish female respondents and may be applied to indicate individuals characterized by the risk of insufficient intake. 

Moreover, ZINC-FFQ may be adjusted for other populations from European countries, due to the lack of the available validated brief questionnaire enabling rapid assessment of the zinc intake for them. The further studies of the ZINC-FFQ are needed in order to indicate the redundant food items, to improve the questionnaire.

## Figures and Tables

**Table 1:** The designed ZINC-food frequency questionnaire (FFQ).

Group of Products	Products	Serving Size	Number of Servings Per Week *
Meat and offals	Pork	100 g (palm of small hand)	
	Poultry		
	Beef		
	Veal		
	Lamb		
	Veal liver		
	Pork and beef liver		
	Other offals		
Cold cuts	Poultry cold cuts	15 g (thin slice of ham, 3 slices of sausage, 1/3 of wiener)	
	Dry sausages		
	Ham, loin, gammon, other sausages		
	Wieners, spam, pate		
Eggs		50 g (1 egg)	
Fish and fish products	Eel, herring, sardine	100 g (palm of small hand)	
	Other fish		
	Fish products from herrings, sprats, sardines	50 g (3 sprats, 1 rollmop)	
	Other fish products	50 g (half of a small can of tuna)	
Milk, dairy beverages, cream		300 g (large glass)	
Cheeses	Emmenthaler, gouda, cheddar, tilsiter, trappist, smoked cheeses	20 g (thin slice)	
	Parmesan, camembert, Roquefort, brie, edam cheese		
	Cottage cheese, curd cheese	30 g (thin slice, tablespoon)	
	Fromage frais, dairy desserts	150 g (packaging)	
	Feta, processed cheese	25 g (slice, triangle serving)	
Breads	Wholemeal, dark breads, breads with grains, graham, pumpernickel bread	30 g (slice, half of a roll)	
	White bread, wheat bread, baguette, rolls, toast bread, confectionery bread		
	Crispbread	10 g (1 slice)	
Other cereal products	Pasta	100 g of cooked (1 glass)	
	Rice		
	Buckwheat, millet		
	Other groats		
	Corn flakes	10 g (1 tablespoon)	
	Other cereals		
	Wheat bran	5 g (tablespoon)	
	Wheat germ		
	Graham flour	10 g (1 tablespoon)	
	Other flours		
Fruits		100 g (half of a glass)	
Vegetables	Legumes	100 g (1 glass of leafy vegetables, half of a glass of the other)	
	Other vegetables		
Potatoes		50 g (2 tablespoons of puree)	
Nuts and seeds	Pumpkin and flax seeds	30 g (handful)	
	Pistachios, coconuts, coconut shreds		
	Other nuts and seeds		
Chocolate products	Cocoa	10 g (1 tablespoon)	
	Plain chocolate	20 g (3–4 chocolate bar squares)	
	Other chocolates and chocolate bars		

* Column to be completed by the respondent. ZINC = Zinc INtake Calculation; FFQ = Food Frequency Questionnaire.

**Table 2:** The designed ZINC**-**FFQ calculation key.

Group of Products	Products	Serving Size	Zinc Content (mg)
Meat and offals	Pork	100 g (palm of small hand)	2.54
	Poultry		1.68
	Beef		3.24
	Veal		2.44
	Lamb		3.09
	Veal liver		8.40
	Pork and beef liver		8.31
	Other offals		2.27
Cold cuts	Poultry cold cuts	15 g (thin slice of ham, 3 slices of sausage, 1/3 of wiener)	0.18
	Dry sausages		0.49
	Ham, loin, gammon, other sausages		0.32
	Wieners, spam, pate		0.21
Eggs		50 g (1 egg)	0.88
Fish and fish products	Eel, herring, sardine	100 g (palm of small hand)	1.12
	Other fish		0.54
	Fish products from herrings, sprats, sardines	50 g (3 sprats, 1 rollmop)	0.62
	Other fish products	50 g (half of a small can of tuna)	0.28
Milk, dairy beverages, cream		300 g (large glass)	1.05
Cheeses	Emmenthaler, gouda, cheddar, tilsiter, trappist, smoked cheeses	20 g (thin slice)	0.77
	Parmesan, camembert, Roquefort, brie, edam cheese		0.52
	Cottage cheese, curd cheese	30 g (thin slice, tablespoon)	0.30
	Fromage frais, dairy desserts	150 g (packaging)	0.92
	Feta, processed cheese	25 g (slice, triangle serving)	0.50
Breads	Wholemeal, dark breads, breads with grains, graham, pumpernickel bread	30 g (slice, half of a roll)	0.66
	White bread, wheat bread, baguette, rolls, toast bread, confectionery bread		0.31
	Crispbread	10 g (1 slice)	0.41
Other cereal products	Pasta	100 g of cooked (1 glass)	0.89
	Rice		1.21
	Buckwheat, millet		3.45
	Other groats		0.90
	Corn flakes	10 g (1 tablespoon)	0.01
	Other cereals		0.13
	Wheat bran	5 g (tablespoon)	0.44
	Wheat germ		0.75
	Graham flour	10 g (1 tablespoon)	0.33
	Other flours		0.08
Fruits		100 g (half of a glass)	0.17
Vegetables	Legumes	100 g (1 glass of leafy vegetables, half of a glass of the other)	1.61
	Other vegetables		0.45
Potatoes		50 g (2 tablespoons of puree)	0.17
Nuts and seeds	Pumpkin and flax seeds	30 g (handful)	2.29
	Pistachios, coconuts, coconut shreds		0.27
	Other nuts and seeds		0.87
Chocolate products	Cocoa	10 g (1 tablespoon)	0.66
	Plain chocolate	20 g (3–4 chocolate bar squares)	0.43
	Other chocolates and chocolate bars		0.22

ZINC-FFQ = Zinc INtake Calculation—Food Frequency Questionnaire.

**Table 3:** The observed zinc intake and the assessment of its adequacy.

Parameters		3-Day Dietary Record	ZINC-FFQ1	ZINC-FFQ2
Mean ± standard deviation (mg)		8.55 ± 3.42	8.61 ± 3.23	8.98 ± 3.45
Median (mg)		8.09 *	8.30 *	8.65 *
Minimum (mg)		3.17	3.00	2.77
Maximum (mg)		22.42	20.97	22.05
Individuals characterized by adequate intake in comparison with EAR level [21]	*n*	69	61	66
	(%)	76.67	67.78	73.33
Individuals characterized by inadequate intake in comparison with EAR level [21]	*n*	21	29	24
	(%)	23.33	32.22	26.67

* Distribution different than normal (Shapiro–Wilk test—*p* ≤ 0.05). ZINC-FFQ1 = first food frequency questionnaire. ZINC-FFQ2 = second food frequency questionnaire. EAR = Estimated Average Requirement.

**Table 4:** The validation of the ZINC-FFQ including tertiles distribution, weighted *κ* statistic and adequacy assessment.

Parameters			ZINC-FFQ1 vs. 3-Day Dietary Record	ZINC-FFQ2 vs. 3-Day Dietary Record	ZINC-FFQ1 vs. ZINC-FFQ2
Individuals classified into the same tertile		*n*	39	42	65
		%	43.33	46.67	72.22
Individuals misclassified (classified into opposite tertiles)		*n*	15	18	3
		%	16.67	20.00	3.33
Weighted *κ* statistic			0.175	0.175	0.65
Individuals of the	same zinc intake adequacy category	*n*	60	59	75
		%	66.67	65.56	83.33
	conflicting zinc intake adequacy category	*n*	30	31	15
		%	33.33	34.44	16.67

ZINC-FFQ1 = first food frequency questionnaire. ZINC-FFQ2 = second food frequency questionnaire.
